# Large-scale Growth and Simultaneous Doping of Molybdenum Disulfide Nanosheets

**DOI:** 10.1038/srep24054

**Published:** 2016-04-05

**Authors:** Seong Jun Kim, Min-A Kang, Sung Ho Kim, Youngbum Lee, Wooseok Song, Sung Myung, Sun Sook Lee, Jongsun Lim, Ki-Seok An

**Affiliations:** 1 Thin Film Materials Research Center, Korea Research Institute of Chemical Technology (KRICT), Daejeon 305-600, Republic of Korea; 2 Nanomaterials Science and Engineering, University of Science and Technology, Daejeon 305-350, Republic of Korea; 3 Chemical Convergence Materials, University of Science and Technology, Daejeon 305-350, Republic of Korea

## Abstract

A facile method that uses chemical vapor deposition (CVD) for the simultaneous growth and doping of large-scale molybdenum disulfide (MoS_2_) nanosheets was developed. We employed metalloporphyrin as a seeding promoter layer for the uniform growth of MoS_2_ nanosheets. Here, a hybrid deposition system that combines thermal evaporation and atomic layer deposition (ALD) was utilized to prepare the promoter. The doping effect of the promoter was verified by X-ray photoelectron spectroscopy and Raman spectroscopy. In addition, the carrier density of the MoS_2_ nanosheets was manipulated by adjusting the thickness of the metalloporphyrin promoter layers, which allowed the electrical conductivity in MoS_2_ to be manipulated.

Transition metal dichalcogenides (TMDs) have attracted a great deal of attention in recent years due to their great potential in various fields including microelectronics, flexible devices, lithium batteries, and optoelectronics^1^,^2^,^3^,^4^,^5^,^6^. Among two-dimensional (2D) TMDs, molybdenum disulfide (MoS_2_), which has a large band gap (1.8 eV), is a suitable material for 2D logic devices and integrated circuits. However, there are two crucial requirements for application in MoS_2_-based nanoelectronics: (i) the synthesis of large-area MoS_2_ nanosheets of high quality and (ii) reliable control of the carrier density in MoS_2_ nanosheets via chemical doping. In recent studies, mechanical and chemical exfoliation methods that utilize bulk MoS_2_ have been performed to obtain monolayer MoS_2_^7^,^8^. However, these methods must be improved to enable the large-scale synthesis of monolayer MoS_2_ in order to apply them to flexible nanoelectronics. The synthesis of single-layer MoS_2_ by chemical vapor deposition (CVD) is suitable for large-scale growth of MoS_2_. In this process, MoS_2_ nanosheets have generally been obtained by sulfurization reactions of deposited molybdenum or molybdenum oxide^9^,^10^. Recently, various seeding promoters have been employed for the synthesis of large-scale MoS_2_ nanosheets^11^. However, obtaining a facile synthesis methodology for high-quality MoS_2_ nanosheets with a uniform thickness still remains as a challenge. In addition, many approaches for the chemical doping of MoS_2_ nanosheets have been reported in order to allow for the manipulation of the carrier density in MoS_2_; the various dopants that have been used include K atoms, Au nanoparticles (NPs), and aromatic molecules^12^,^13^,^14^,^15^,^16^,^17^.

In this study, we present a new approach for the simultaneous large-scale synthesis and doping of MoS_2_ nanosheets by applying metalloporphyrin layers as a seeding promoter layer. As shown in Fig. 1, a H_2_TPP thin layer was first formed on a SiO_2_ substrate by thermal evaporation. Subsequent metalation of the H_2_TPP films was carried out for the formation of different metalloporphyrins such as Al(III)-tetraphenyl porphyrin (Al(III)TPP) or Zn(II) meso-tetra(4-hydroxyphenyl) porphyrin (Zn(II)THPP). Here, the metallic atoms in metalloporphyrin were used as dopants for the MoS_2_ nanosheets. For the preparation of metalloporphyrins, including Al(III)TPP and Zn(II)THPP, we utilized an alternative synthetic method consisting of the thermal evaporation of organic molecules and the metalation process for the formation of metalloporphyrin (Fig. S1 in the Supporting Information). Conventional thermal CVD (TCVD) was subsequently carried out for the simultaneous large-scale synthesis and doping of MoS_2_ nanosheets on the metalloporphyrin layers. These MoS_2_ nanosheets were transferred to arbitrary substrates such as SiO_2_ and polyethylene terephthalate (PET) substrates. Finally, Cr/Au electrodes were deposited onto the MoS_2_ nanosheets to complete the MoS_2_-based electrical devices.

## Results

### Preparation of Al(III)TPP and Zn(II)THPP promoter layers

A hybrid deposition system that combines thermal evaporation and a metalation process was utilized for the synthesis of the metalloporphyrin promoter (Fig. S1 in the Supporting Information). Here, H_2_TPP was employed as the main organic source and trimethylaluminum (TMA) and diethylzinc (DEZ) were used as the precursors for the Al(III)TPP and Zn(II)THPP promoters, respectively. In the case of the Al(III)TPP promoter, the formation mechanism of metalloporphyrin (i.e., Al(III)TPP) can be explained by a two-step reaction (Fig. S2(a) in the Supporting Information). In the first step, H_2_TPP molecules were evaporated on a solid substrate via thermal evaporation. Next, the TMA precursor was introduced into the reactor where it reacted with H_2_TPP; in this process, two pyrrolic nitrogen atoms in the center of H_2_TPP coordinated with an Al atom of TMA during metalation (H_2_TPP + Al(CH_3_)_3_ → Al(III)TPP (CH_3_) + 2CH_4_↑). Two methyl groups (−CH_3_) of TMA and the hydrogen of pyrrolic nitrogen combined to form methane (CH_4_) as a byproduct; this was removed by a purging process. Finally, the Al atom that was bonded with two pyrrolic nitrogens was coordinated by two iminic nitrogen atoms in the center of H_2_TPP. Thereafter, the thicknesses of the Al(III)TPP layers were controlled by repeating this two-step reaction (i.e., H_2_TPP evaporation and Al metalation). Importantly, the electrical properties of the MoS_2_ nanosheets can be easily and accurately controlled by adjusting the type and density of the dopants of the MoS_2_ layers. For the formation of the Zn(II)THPP promoter, the DEZ precursor reacts chemically with THPP molecules on the solid substrate via a gas phase metalation process. Here, four Zn atoms of DEZ interacted with hydroxyl groups on the meso position of porphyrin. Additionally, two pyrrolic and iminic nitrogen atoms in the center of the porphyrin molecule coordinated with a Zn atom of DEZ during the metalation process. Afterwards, vapor-phase ethyl groups in DEZ were eliminated completely in the form of C_2_H_6_ as byproducts. (THPP + 6Zn(CH_2_CH_3_)_2_ → Zn(II)THPP + 6C_2_H_6_↑) (Fig. 3S of the Supporting Information).

### CVD growth of MoS_2_ nanosheets on Al(III)TPP and Zn(II)THPP promoter layers

Conventional TCVD was utilized for the simultaneous large-scale synthesis and doping of MoS_2_ nanosheets on metalloporphyrin promoter layers. Here, the thickness of the metalloporphyrin promoter layers was manipulated by adjusting the number of metalloporphyrin coating cycles to tune the carrier density of the MoS_2_ nanosheets. A metalloporphyrin promoter layer, on a solid substrate, was placed onto an alumina boat in the center of the reactor, as illustrated in Fig. S4 of the Supporting Information. A Mo solution was prepared by dissolving 0.1 M ammonium heptamolybdate in distilled water; this was subsequently spin-coated onto SiO_2_ substrates. Sulfur powder, which was used as the sulfur source, was located upstream in the reactor. MoS_2_ nanosheets were grown at 600 °C while introducing Ar gas for 5 min.

**Figure 2:**
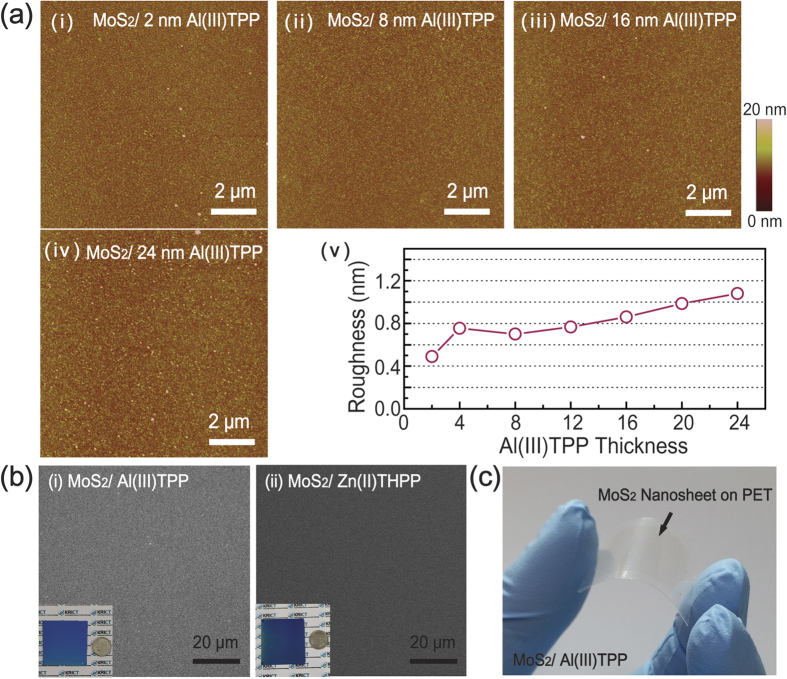
Large-scale growth of MoS_2_ nanosheets using Al(III)TPP and Zn(II)THPP seeding promoters. (**a**) AFM images of the MoS_2_ films on metalloporphyrin (Al(III)TPP) as a function of Al(III)TPP thickness ((i) 2, (ii) 8, (iii) 16, and (iv) 24 nm). (v) Plots of the RMS roughness of the MoS_2_ films on the Al(III)TPP promoter as a function of the number of Al(III)TPP layers. (**b**) An SEM image of MoS_2_ nanosheets on metalloporphyrin promoters ((i) Al(III)TPP and (ii) Zn(II)THPP). The inset shows a photograph of MoS_2_ nanosheets. (**c**) Photograph of MoS_2_ nanosheets transferred onto a flexible substrate (PET) by PMMA-assisted wet transfer.

We investigated the evolution of the surface morphologies of MoS_2_ nanosheets formed on Al(III)TPP promoters with a variety of thicknesses (i.e., 2, 8, 16, and 24 nm thick), as shown in Fig. 2(a). As the Al(III)TPP thickness increased, the root mean square (RMS) roughness of the MoS_2_ nanosheets increased. The RMS roughness of the nanosheets was approximately 1.0 nm, indicating that our MoS_2_ nanosheets have an ultra-flat surface. As shown in Fig. 2(b), representative scanning electron microscopy (SEM) images of MoS_2_ nanosheets grown on (i) Al(III)TPP and (ii) Zn(II)THPP promoter layers confirmed that continuous MoS_2_ nanosheets with a uniform thickness were synthesized on the promoter layers; this result is dissimilar from previous results, including the synthesis of triangular MoS_2_ flakes^11^. The photograph in the inset of Fig. 2(b) exhibits the excellent uniformity of the MoS_2_ nanosheets on (i) Al(III)TPP and (ii) Zn(II)THPP over large areas (4 × 4 cm^2^).

In addition, we also applied a poly(methylmethacrylate) (PMMA)-assisted wet transfer method for the fabrication of MoS_2_-based flexible devices^18^. Figure 2(c) shows a photograph of MoS_2_ nanosheets transferred onto polyethylene terephthalate (PET). Here, PMMA was spin-coated onto the MoS_2_ surface, and a PMMA-coated MoS_2_/SiO_2_ substrate was placed in 4 M NaOH in DI water. After the SiO_2_ layer was completely etched away, the PMMA-coated MoS_2_ nanosheets were transferred to the target substrates. Finally, the PMMA layer was eliminated by rinsing with acetone, and the remaining MoS_2_ nanosheets were rinsed with DI water. Raman spectroscopy confirmed that the transferred MoS_2_ nanosheets on the target substrate were stable under these transferring and etching processes. It should be noted that our method allows us to synthesize large-scale, two-dimensional MoS_2_ nanosheets with uniform thickness.

### Raman characterization of MoS_2_ nanosheets on Al(III)TPP and Zn(II)THPP layers

**Figure 3:**
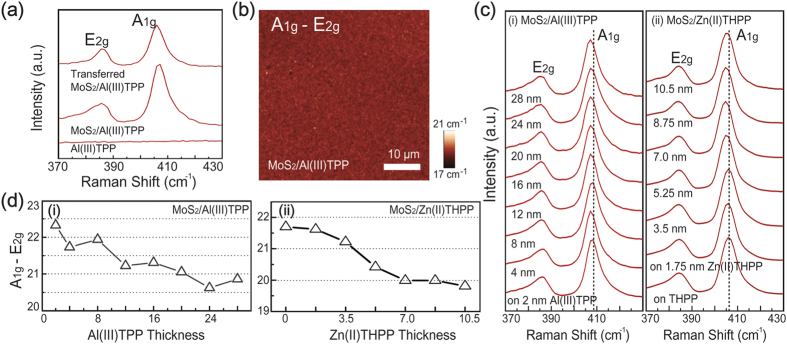
Raman characterization of MoS_2_ nanosheets. (**a**) Raman spectra of Al(III)TPP and MoS_2_ nanosheets on Al(III)TPP promoter layers before and after the PMMA-assisted wet transfer. (**b**) Raman A_1g_-E_2g_ map of the MoS_2_ nanosheets on Al(III)TPP promoter layers. (**c**) Raman spectra of MoS_2_ films grown with various Al(III)TPP and Zn(II)THPP thicknesses. (**d**) The difference between the A_1g_ and E_2g_ Raman modes of the MoS_2_ nanosheets as a function of Al(III)TPP and Zn(II)THPP thickness.

Raman spectroscopy, which is a powerful nondestructive characterization tool, was utilized to study the crystalline structures of the MoS_2_ nanosheets grown on metalloporphyrin. In the Raman spectra of the MoS_2_ sheets on the initial substrate and the transferred MoS_2_ film, two prominent peaks at ~408 cm^−1^ and ~386 cm^−1^, which originated from the out-of-plane vibration mode (A_1g_ mode) of sulfur atoms and the in-plane vibration mode (E_2g_ mode) of molybdenum and sulfur atoms, respectively, were observed. These peaks were also present after the transfer process onto the desired substrate (Fig. 3(a)), indicating that the MoS_2_ nanosheets were stable during the wet transfer processes (including wet-etching and the removal of PMMA). In addition, the number of MoS_2_ layers can be identified by analyzing the energy difference between the A_1g_ and E_2g_ modes^10^. Figure 3(b) shows the uniformity of MoS_2_ layers on the Al(III)TPP promoter, as evaluated by Raman mapping. Here, Raman maps of A_1g_-E_2g_ confirmed the excellent uniformity of the number of layers. Figure 3(c,d) show the Raman spectra and the energy difference of the A_1g_ and E_2g_ modes for MoS_2_ nanosheets on Al(III)TPP (or Zn(II)THPP) promoter layers as a function of the number of coating cycles, respectively. These results indicate that bilayer MoS_2_ nanosheets were synthesized with 2–20 nm of the Al(III)TPP (or 0–3.5 nm Zn(II)THPP) promoter and monolayer MoS_2_ was obtained with 24–28 nm of the Al(III)TPP (or 5.25–10.5 nm Zn(II)THPP) promoter. Remarkably, this result showed that mono- and bi-layer MoS_2_ nanosheets were fabricated by adjusting the thickness of the Al(III)TPP (or Zn(II)THPP) promoter layers. The UV-Vis absorption spectra of the MoS_2_ nanosheets that were transferred onto PET revealed two prominent absorption peaks (A1 and B1 exciton transitions) at 655 nm (1.89 eV) and 610 nm (2.03 eV), which is similar to the results of a previous study^19^. The intensity of these two peaks decreased as the number of coating cycles of Al(III)TPP promoter layers increased, indicating that the layer number of MoS_2_ sheets decreased when using a promoter layer with a thickness between 20 and 30 nm (S5 in the Supporting Information). This result is in good agreement with the Raman analysis shown in Fig. S5 in the Supporting Information.

### Electrical properties of MoS_2_ nanosheets synthesized on Al(III)TPP and Zn(II)THPP promoters

**Figure 4:**
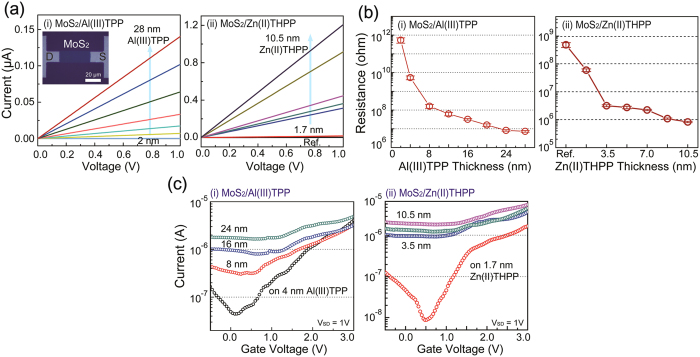
Electrical properties of MoS_2_ nanosheets synthesized on metalloporphyrin promoters. (**a**) Output characteristics (I_DS_-V_DS_) of MoS_2_ nanosheets grown on (i) Al(III)TPP and (ii) Zn(II)THPP promoters with various thicknesses; these were mediated by adjusting the number of coating cycles. (**b**) The extracted resistance of MoS_2_ nanosheets as a function of the thickness of (i) Al(III)TPP and (ii) Zn(III)THPP promoters. (**c**) Transfer characteristics (I_DS_-V_G_) of MoS_2_ nanosheets grown on (i) Al(III)TPP and (ii) Zn(II)THPP promoters with various thicknesses (mediated).

In order to characterize the electrical properties of the MoS_2_ nanosheets synthesized on metalloporphyrin promoters with various thicknesses, MoS_2_-based devices were fabricated via conventional microfabrication processes (e.g., photo-lithography) and thermal evaporation was used to make the electrodes. Here, the MoS_2_ channel length and width were 100 and 40 mm, respectively, and Cr/Au was used as the source and drain electrodes. The output characteristics (I_DS_-V_DS_) of the nanoscale devices made with MoS_2_ on Al(III)TPP and Zn(II)THPP, with various thicknesses, were measured as a function of the number of coating cycles. These results demonstrate that the electrical conductivity of MoS_2_ gradually increases as the thickness of the Al(III)TPP and Zn(II)THPP promoters increases, as shown in Fig. 4(a). Figure 4(b) also shows the extracted resistance of the MoS_2_ nanosheets grown on Al(III)TPP and Zn(II)THPP promoters with various thicknesses. These results indicate that the resistance of MoS_2_ nanosheets remains unchanged when a promoter layer above a specific thickness is used during the growth processes. Additionally, an electrical double-layer transistor based on MoS_2_ on metalloporphyrin was demonstrated by using 1-butyl-3-methylimidazolium (BmimPF_6_) as an ionic liquid gate^20^,^21^. Here, the length and width of the channel between the Cr/Au electrodes were 100 and 40 μm, respectively. Figure 4(c) shows the representative transfer characteristics (I_DS_-V_G_) at V_DS_ = 1 V for MoS_2_-based transistors grown on metalloporphyrin. To analyze the doping effect caused by the metalloporphyrin promoter, MoS_2_ sheets grown on metalloporphyrin layers with different thickness were prepared. In the case of a MoS_2_ layer on a metal-free *p*-THPP promoter, n-type semiconducting behavior with an on-off ratio of 10^2^ and a threshold voltage of about 0.5 V was observed, as shown in Fig. 4(c). When using metalloporphyrin promoters, such as Al(III)TPP and Zn(II)THPP, the on-off ratio decreased. For example, MoS_2_-based transistors on 28-nm-thick Al(III)TPP and 10.5-nm-thick Zn(II)THPP promoters exhibited metallic behavior, which confirmed the doping effect of metalloporphyrin. However, as shown in Figure S9 in Supporting Information, the electron mobility of the MoS_2_-based transistors decreased as increasing promoter thickness, since the dopant of metalloporphyrin produced impurity scattering in the MoS_2_ nanosheets.

## Discussion

### X-ray photoelectron spectroscopy (XPS) of MoS_2_ nanosheets synthesized on Al(III)TPP and Zn(II)THPP promoter layers

**Figure 5:**
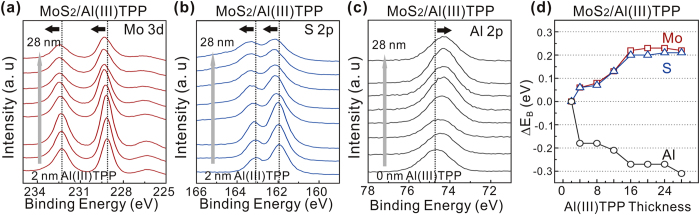
X-ray photoelectron spectroscopy (XPS) of MoS_2_ nanosheets synthesized on Al(III)TPP promoter layers. (**a**) Mo 3d, (**b**) S 2p, and (**c**) Al 2p core level spectra of MoS_2_ nanosheets using Al(III)TPP promoters with various thicknesses (2–28 nm). (**d**) The binding energy shifts (ΔE_B_) of Mo 3d, S 2p, and Al 2p of MoS_2_ nanosheets as a function of Al(III)TPP thickness.

In order to analyze the mechanism that causes the increase of MoS_2_ nanosheets on the metalloporphyrin, XPS was utilized. First, we prepared MoS_2_ sheets on the Al(III)TPP promoter with various thicknesses. Figure 5(a–c) exhibit the Mo 3d, S 2p, and Al 2p core level spectra obtained from MoS_2_ nanosheets synthesized on Al(III)TPP promoter layers with various thicknesses, which were obtained by adjusting the number of coating cycles. Interestingly, the Mo 3d and S 2p peaks shifted to higher binding energies whereas the Al 2p peak shifted to a lower binding energy as the thickness of the Al(III)TPP promoter layer was increased (Fig. 5(d)). This behavior can be understood in terms of the effect of n-type doping of the Al nanoparticles (AlNPs) extracted from the Al(III)TPP promoter; this effect originates from electron charge transfer from Al (4.08 eV) to MoS_2_ (4.7 eV), which is induced by differences in the work function^22^,^23^. When the TCVD was heated to the target temperature during MoS_2_ synthesis, the thickness of the Al(III)TPP promoter layers decreased because of thermal evaporation. In this process, the carbon component was partially evaporated, whereas Al remained, as shown in Fig. S6 in the Supporting Information. This result was supported by the fact that the intensity of the Al 2p peak obtained from Al(III)TPP increased and the intensity of the C 1s peak decreased after annealing at 900 °C, as shown in Fig. S6 in the Supporting Information. Consequently, the remaining AlNPs electrically interacted with the upper MoS_2_ nanosheets and acted as n-type dopants. Furthermore, no noticeable change in the binding energy of the C 1s peak was observed, as shown in Fig. S7 of the Supporting Information. This indicates that there is no electrical interaction between the thinned Al(III)TPP promoter layers and the MoS_2_ nanosheets. Furthermore, in the case of the Zn(II)THPP promoter, the XPS results also indicated that the conductance increase in MoS_2_ nanosheets resulted from the doping effect of Zn nanoparticles. This is similar to what occurred in Al(III)TPP (Fig. S8 in the Supporting Information).

In summary, we demonstrated an innovative method for the simultaneous large-scale synthesis and doping of MoS_2_ nanosheets using metalloporphyrin promoters such as Al(III)TPP and Zn(II)THPP. We first prepared metalloporphyrin promoters using a hybrid deposition system that utilized thermal evaporation and metalation. The structural and electrical characteristics of MoS_2_ nanosheets on metalloporphyrin promoters with various thicknesses were systematically investigated. In addition, the effect of n-type doping of metallic nanoparticles extracted from the promoter was explored through XPS analysis. Our facile approach may pave the way for the large-scale synthesis of high-quality MoS_2_ nanosheets and the fabrication of MoS_2_ nanosheets with controlled electrical conductivity for advanced two-dimensional nanoelectronic applications.

## Methods

### Preparation of the Al(III)TPP (or Zn(II)THPP) promoter layer

Al(III)TPP (or Zn(II)THPP) thin films used as a promoter layer were formed on SiO_2_ (300 nm)/Si (100) using a hybrid deposition system that combined thermal evaporation and ALD, as depicted in Fig. 2a. The following growth process was implemented. First, the sample was placed on a rotatable holder in the main chamber. Then, 5, 10, 15, 20-tetraphenylporphyrin (H_2_TPP) (or 5, 10, 15, 20-​tetrakis(4-​hydroxyphenyl)-21*H*, 23*H*-porphyrin (THPP)) was deposited on the SiO_2_ surface by opening the main gate valve and facing the sample downward. After the deposition of 0.35-nm-thick H_2_TPP (or THPP) films, the main gate valve was closed and the sample holder was rotated upward. Al(III)TPP (or Zn(II)THPP) films were formed onto the monolayer H_2_TPP (or THPP) films by introducing trimethyl aluminum (TMA) (or diethyl zinc (DEZ)) under 1.3 × 10^−1^ Torr for 5 s (or 1.4 × 10^−1^ Torr for 40 s). Finally, a purge process was conducted by flowing liquid nitrogen gas at 500 sccm for 30 s. Al(III)TPP (or Zn(II)THPP) films were eventually formed by repeating this growth cycle. The thicknesses of the Al(III)TPP (or Zn(II)THPP) films were controlled by adjusting the number of coating cycles from 5 to 70 cycles (or from 0 to 30 cycles).

### CVD growth of MoS_2_ nanosheets on Al(III)TPP (or Zn(II)THPP) promoter layers

The Mo solution was prepared by dissolving 0.1 M ammonium heptamolybdate (Fluca, 99%) in 10 mL of distilled water. This solution was subsequently coated onto UV-treated SiO_2_ (300 nm) substrates by spin-coating at 2000 rpm for 30 s. 0.1 g of sulfur powder (SAMCHUN, 98.0%), which was used as the sulfur source, was located upstream in the reactor. The distance between the sulfur and Mo sources was 19 cm. AlNP (or ZnNP)-doped MoS_2_ nanosheets were synthesized at 600 °C under ~1 Torr while introducing Ar (500 sccm) for 5 min.

## Additional Information

**How to cite this article**: Kim, S. J. *et al*. Large-scale Growth and Simultaneous Doping of Molybdenum Disulfide Nanosheets. *Sci. Rep.* **6**, 24054; doi: 10.1038/srep24054 (2016).

## Supplementary Material

Supplementary Information

## Figures and Tables

**Figure 1:**
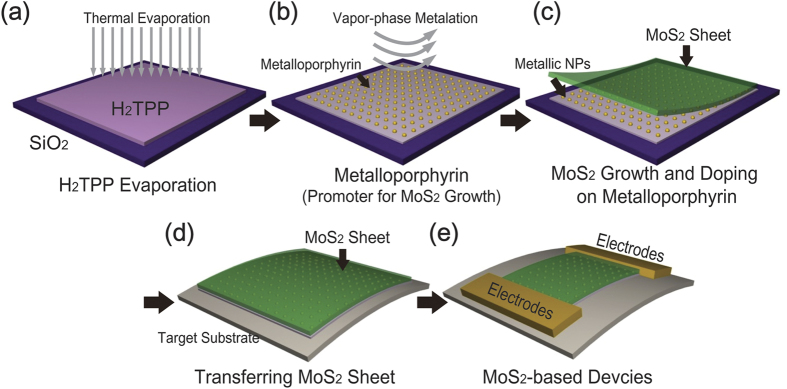
Schematic of the synthesis process of metalloporphyrin and MoS_2_ nanosheets. The procedure utilizes a hybrid deposition system combining (**a**) thermal evaporation and (**b**) a metalation process. (**c**) Growth of MoS_2_ nanosheets on a seeding promoter layer. (**d**) The transfer of MoS_2_ films on metalloporphyrin toward target substrates. (**e**) The fabrication of the MoS_2_-based device.
