# Supplementary material for: Large-scale Growth and Simultaneous Doping of Molybdenum Disulfide Nanosheets
Source: Sci Rep. 2016 Apr 5;6:24054. doi: 10.1038/srep24054 (PMC4820707; doi:10.1038/srep24054)
Supplement: Supplementary Information [file srep24054-s1.pdf]

Large-scale Growth and Simultaneous Doping of Molybdenum Disulfide Nanosheets

Seong Jun Kim[1,2], Min-A Kang[1], Sung Ho Kim[1], Youngbum Lee[1], Wooseok Song[1], Sung Myung[1], Sun Sook Lee[1], Jongsun Lim[1], and Ki-Seok An,[1,3]**

[1]Thin Film Materials Research Center, Korea Research Institute of Chemical Technology (KRICT), Daejeon 305-600, Republic of Korea.

[2]Nanomaterials Science and Engineering, University of Science and Technology, Daejeon 305-350, Republic of Korea.

[3]Chemical Convergence Materials, University of Science and Technology, Daejeon 305-350, Republic of Korea.

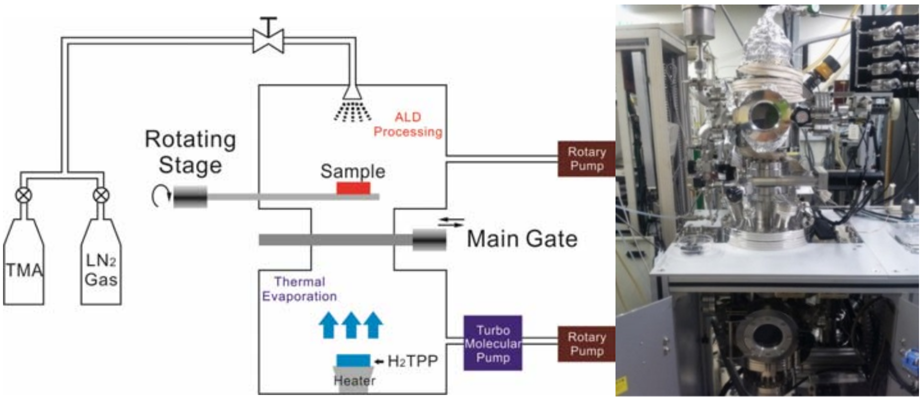

Fig. S1. Schematic representation of the hybrid deposition system combining thermal evaporation (bottom) and ALD (top).

Growth mechanism of Al(III)TPP promoter layer. This growth mechanism of Al(III)TPP film is supported by the X-ray photoelectron spectroscopy (XPS) results shown in Fig. S2(b). The XPS spectra were acquired with a normal emission geometry using conventional monochromatic Al Kα radiation (hv = 1,486.6 eV). The pass energy was 50.0 eV and the spectra were deconvoluted by a standard nonlinear least squares fitting procedure using Voigt functions. Fig. S2(b-i) exhibits the N 1s core level spectra of the H_2TPP films, where the pyrrolic nitrogen (-NH-) at 399.9 eV and iminic nitrogen (=N-) at 397.8 eV were observed.[S1] With increasing TMA exposure time, the intensities of the pyrrolic and iminic nitrogen peaks decreased and that of the metalloporphyrin complex-related peak (yellow) at 398.4 eV appeared. We investigated the relationship between the areas of the -NH- and Al 2p peaks and the TMA exposure time. The area of the -NH- peak decreased significantly with increasing TMA exposure time up to 5 s, after which the area of the -NH- peaks showed saturation behavior, as shown in Fig. S2(c). Hence, we adopted a TMA exposure time of 5 s for the formation of the Al(III)TPP promoter layers.

[Ref S1] R. Gonzalez-Moreno, C. Sanchez-Sanchez, M. Trelka, R. Otero, A. Cossaro, A. Verdini, L. Floreano, M. Ruiz-Bermejo, A. Garcia-Lekue, J. A. Martin-Gago, C. Rogero, *J. Phys. Chem. C*, 2011, **115**, 6849.

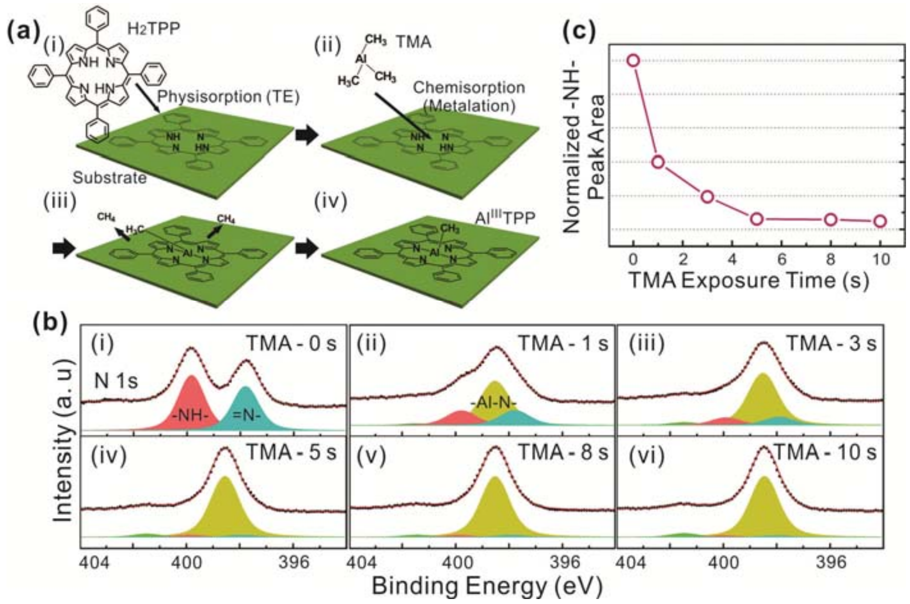

Fig. S2. Preparation of Al(III)TPP layer. (a) Schematic representation of the formation mechanism of Al(III)TPP as a seeding promoter layer. (b) XPS spectra of the N 1s core level for H_2TPP and Al(III)TPP nanosheets formed by introducing the TMA precursor for 1, 3, 5, 8 and 10 s. (c) Plots of the normalized -NH- peak area extracted from the survey and N 1s core level spectra as a function of the TMA precursor exposure time (1, 3, 5, 8, and 10 s).

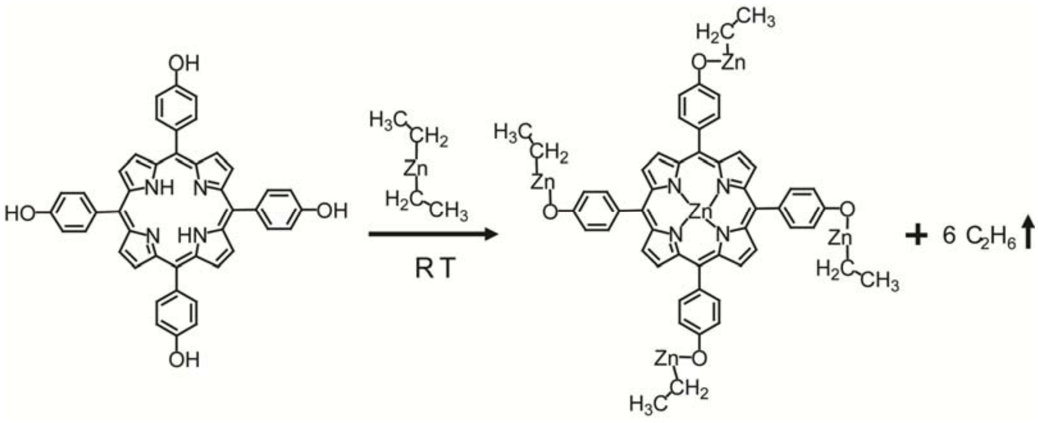

Fig. S3. Surface reaction for Zn(II)THPP promoter layer.

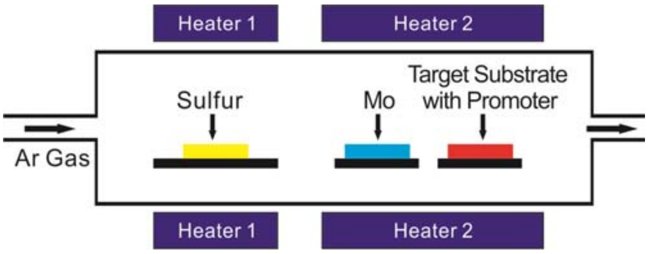

Fig. S4. A schematic diagram of the TCVD system used for simultaneous large-scale synthesis and doping of MoS_2 nanosheets on metalloporphyrin promoter layers.

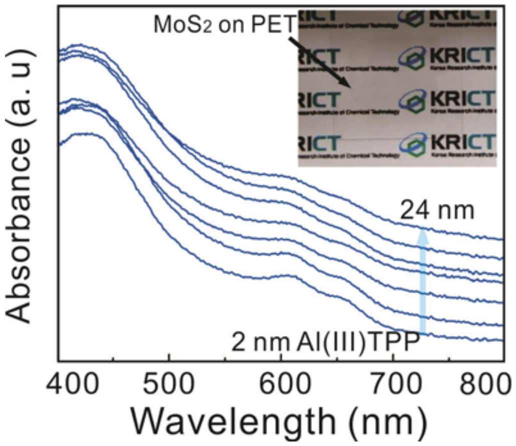

Fig. S5. UV-Vis absorption spectra of MoS_2 nanosheets grown on Al(III)TPP promoter layers with various thicknesses (2-24 nm).

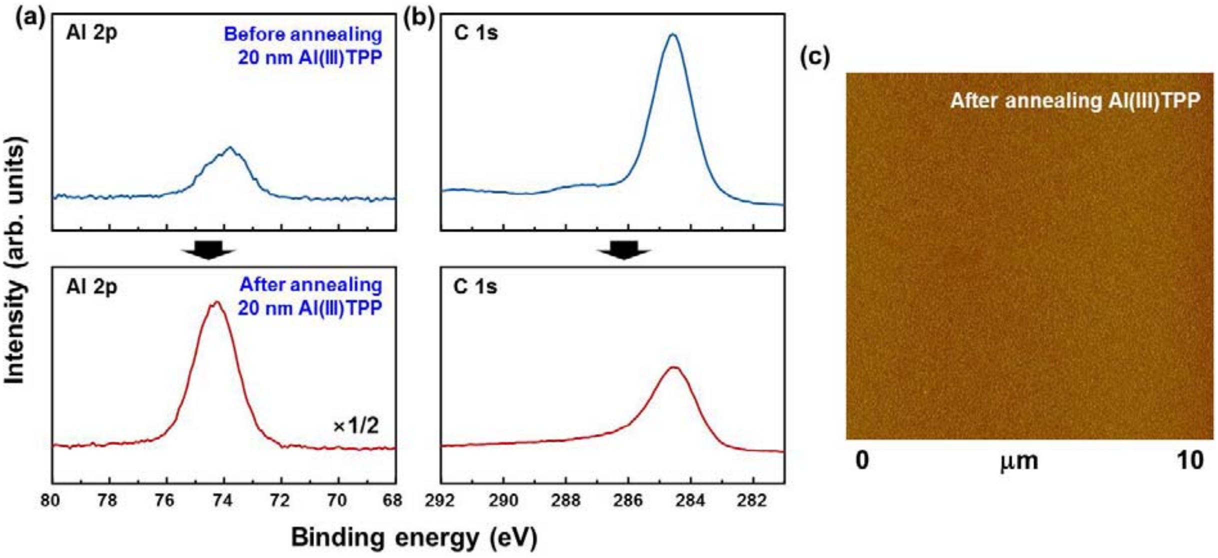

Fig. S6. XPS spectra of (a) Al 2p and (b) C 1s core level in Al(III)TPP before and after annealing. (c) AFM topographic image of an Al(III)TPP layer after annealing.

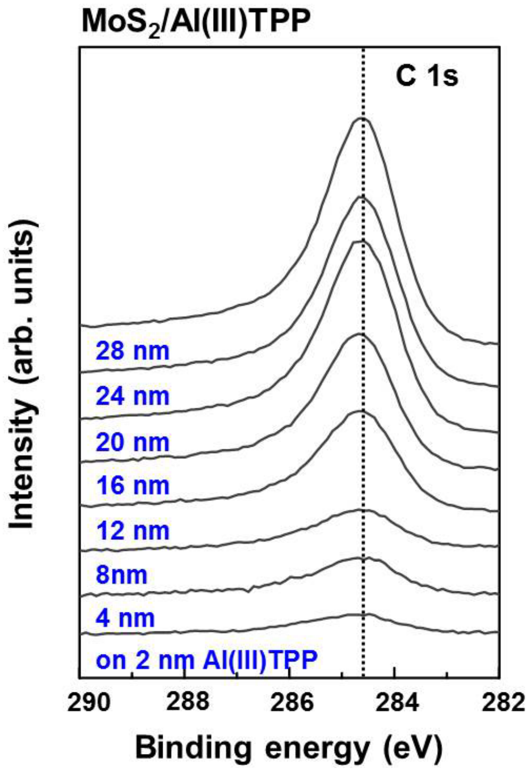

Fig. S7. XPS C 1s core level spectra of a MoS_2 nanosheet on Al(III)TPP promoter layers.

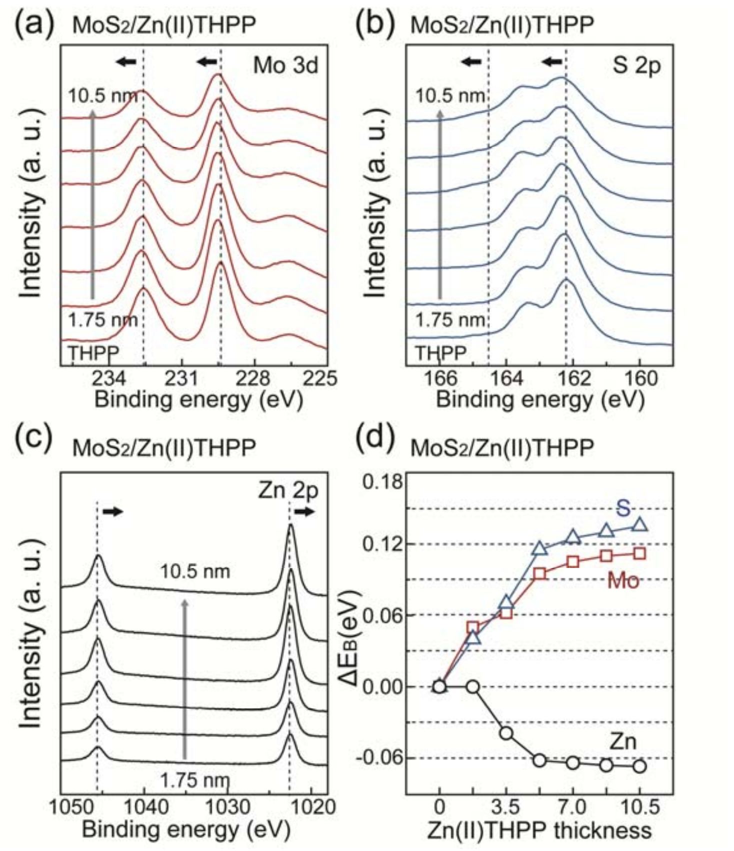

Fig. S8. XPS of MoS_2 nanosheets synthesized on Zn(II)THPP promoter layers: (f) Mo 3d, (g) S 2p, and (h) Zn 2p core level spectra of MoS_2 nanosheets using Zn(II)THPP formed by adjusting the number of synthetic cycles. (i) The binding energy shift (ΔE_B) of Mo 3d, S 2p, and Zn 2p of the MoS_2 nanosheets as a function of Zn(II)THPP thickness.

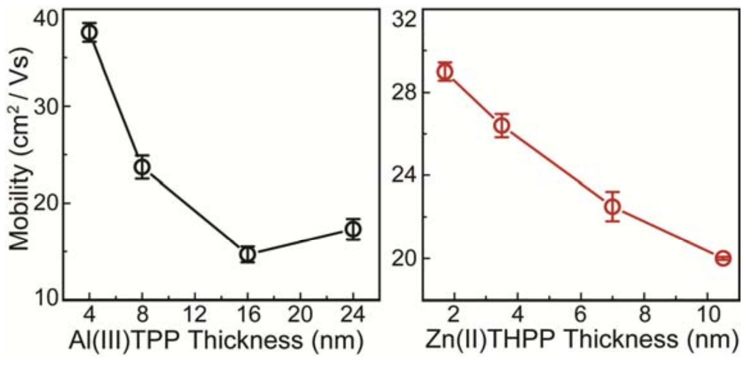

Fig. S9. The field-effect mobility of the MoS_2-based devices extracted as a function of the thickness of Al(III)TPP (left) and Zn(II)THPP (right) promoters.
